# Plaster of Paris in Recent Fractures

**Published:** 1890-07

**Authors:** W. H. Elliott

**Affiliations:** Savannah, Ga.


### PLASTER OF PARIS IN RECENT FRACTURES.*

BY W. H. ELLIOTT, M. D., SAVANNAH, GA.

Unfortunately for mankind, railway accidents frequently occur at points remote from the centers of civilization, and the sufferers are often subjected to serious delay, and the pain of transportation, before their injuries can be properly attended to. Of all classes of injury, cases of fracture, especially of the lower extremity, are among the most difficult to transport with ease and safety. To accomplish this, some temporary support is usually applied to the broken limb, to be superseded by a permanent apparatus after arrival at destination.

Now, as far as the present comfort and future welfare of the patient are concerned, it would be much better if the broken bone could be set at once, and the limb put up in some form of immovable apparatus. This corrects the displacement, reduces to a minimum inflammation and swelling, and places the sufferer in the best condition to bear without pain or injury the journey before him. If properly applied, an apparatus of this kind will also serve for treatment till the termination of the case; thus avoiding a resetting and the risk of displacement.

But, as usually made, the immovable apparatus cannot be safely applied to recent fractures, for the reason that it does not allow for subsequent swelling. This *must* be provided for; because a railway patient is often compelled to travel long distances, without the care of a surgeon, and circumstances may greatly extend the time of a journey.

Under these circumstances, the rule is imperative that no dressing can be applied, which is liable to constrict the limb when swelling comes on.

In any apparatus that entirely encircles the limb, the least amount of swelling will increase the constriction at the swollen

*Read before National Association of Railway Surgeons, May, 1890.

part, and this constriction will, in its turn, increase the swelling, and the vicious process thus begun will go on from bad to worse with accelerating speed till impeded circulation ends in mortification of the limb, and I must add, of the surgeon.

Is there then any form of immovable apparatus that can be applied to recent fractures, which shall be absolutely free from the risk of constriction and mortification? I answer, yes. Plaster of Paris may be so used that it can be put on any fracture, no matter how recent, with perfect safety.

There are two methods of application. One is the well known plaster of Paris *bandage*. You are all familiar with the mode of application. It entirely surrounds the limb, and to make it safe, it must, as soon as the plaster has firmly set, be cut open from one end to the other.

The other method is known as the plaster of Paris *splint*, first brought prominently to the notice of the profession by the late Dr. James L. Little, of New York.

This splint is made of cotton flannel, or stockinet, thoroughly impregnated with a mixture of plaster and water, and accurately fitted while still wet and limp to the injured limb, which it should not encircle for more than four-fifths of its circumference, thus leaving, on any aspect of the limb that the operator pleases, an opening one or two inches wide down throughout the entire length of the splint.

In making the splint, the first requisite is good plaster. This can be had by taking from the center of a fresh barrel. It can be kept for many months, if sealed in tin buckets, and every railway surgeon should keep a supply in his office.

The method of application is as follows:

First envelop the broken limb in cotton wadding, or a thick flannel bandage, which must afterward be cut open. Then cut out two or three pieces of canton flannel, or stockinet, to fit the limb from the extremity to the first joint above the seat of fracture, being careful that the cloth shall not meet around the limb by one or two inches. To avoid disturbing the injured limb, the cloth can be fitted to the sound limb, and the splint reversed in the application. Mix the plaster thoroughly with a little less than its volume of water, till the mixture is smooth and free from lumps. Stir the pieces of cloth successively in the mixture, till they are thoroughly wet ; spread the cloth upon a table, piece upon piece ; smoothing each one with the hand before the next is laid on it. Now lift the splint from the table, and apply it in its flaccid condition to the limb ; the assistants holding the edges of the cloth as close together as possible, to insure coaptation without wrinkles, while the operator binds it closely with an ordinary muslin bandage. Then set the fracture, making the proper manipulations, including extension, and counter-extension, which must be maintained till the plaster is hard. This will usually take about fifteen minutes, more or less, as the mixture is thick or thin.

Then take off the bandage. The injured limb is now lying in an immovable, well-cushioned splint; which, if properly fitted, can be left on till union is complete, for the plaster splint being porous, does not retain the cutaneous excretions. The splint being open its whole length and yielding to any swelling no constriction of the limb can take place, and a simple inspection will show the condition of the circulation, and of the injured parts generally. As soon as the swelling begins to subside, the splint, which is elastic, can be drawn in by a bandage. A little starch will prevent this from slipping loose, and is a convenient mode of reinforcing the splint at any point that may be desired.

The splint can also be taken off and replaced at will. The bearing of this fact is obvious in the treatment of protracted cases, where it may be beneficial to remove the splint every night, for rest and treatment to the wearied tissues, and replace it in the morning for out-door exercise.

The plaster splint holds a position intermediate between the ordinary splints usually applied to recent fractures, and the various forms of permanent apparatus used later on. It differs from the ordinary splint, in that it can retain itself and the broken limb in position, without the use of a bandage, during the critical period of inflammation and swelling; when a retaining bandage keeps the surgeon anxious and ever on the watch. On the other hand, it differs from the immovable apparatus in securing immobility, without the risk of interfering with the circulation.

I trust that further argument is unnecessary, to show the superiority of the plaster splint, to all other methods of treating fractures of the limbs. It is readily and safely applied to recent fractures except those of the forearm. It can be perfectly adapted to the parts, and when once well adjusted, retains its position, and so requires less work and watchfulness from the surgeon.

Lastly, I think it attains the best results. I can bring no statistics to establish this point, but my opionion is based upon twenty years' experience, during which time I have been in the habit of using this splint in recent fractures, sometimes applying it before the patient was moved at all.

I have frequently, by its use, obtained union without shortening in fractures of the femur, a result which I have been unable to attain by any other apparatus.

